# SOCS2 Suppresses Inflammation and Apoptosis during NASH Progression through Limiting NF-κB Activation in Macrophages

**DOI:** 10.7150/ijbs.63889

**Published:** 2021-10-11

**Authors:** Shuo Li, Sheng Han, Kangpeng Jin, Tingting Yu, Han Chen, Xiaoying Zhou, Zhongming Tan, Guoxin Zhang

**Affiliations:** 1 Department of Gastroenterology, the First Affiliated Hospital of Nanjing Medical University, Jiangsu, China.; 2 Hepatobiliary Center, The First Affiliated Hospital of Nanjing Medical University.; 3 Key Laboratory of Liver Transplantation, Chinese Academy of Medical Sciences.; 4 NHC Key Laboratory of Living Donor Liver Transplantation (Nanjing Medical University), Nanjing, Jiangsu Province, China.; 5 Department of General Surgery, The First Affiliated Hospital of Nanjing Medical University, Nanjing, Jiangsu, 210029, People's Republic of China.; 6 The First School of Clinical Medicine, Nanjing Medical University, Nanjing, People's Republic of China.

**Keywords:** Nonalcoholic steatohepatitis, Suppressor of cytokine signaling 2, inflammation, apoptosis, NF-κB

## Abstract

**Background:** Inflammation and apoptosis play a crucial role in the progression of nonalcoholic steatohepatitis (NASH). Suppressor of cytokine signaling 2 (SOCS2) is one of classic negative regulators of cytokine signaling, which has recently been described as anti-inflammatory mediators. However, the role of SOCS2 in macrophages during NASH progression and the relationship among SOCS2, inflammation, apoptosis and NASH is largely unknown. Herein, we aimed to study the function of SOCS2 in NASH progression.

**Methods:** We detected SOCS2 expression in macrophages in human subjects without steatosis, with simple steatosis and with NASH to confirm the relationship between SOCS2 and NASH. Free fatty acids was used to establish stress environment in RAW 264.7 cell lines stably overexpressing or knockdown SOCS2. *In vitro* and vivo assays also performed to study the molecular function of SOCS2 in NASH progression.

**Findings:** Our human samples illustrated that SOCS2 was decreased in macrophages during NASH progression and was negatively correlated to NASH level. Meanwhile, *In vitro* assays showed SOCS2 overexpression in macrophages suppressed inflammation and apoptosis via inhibiting NF-κB signaling pathway, while SOCS2 knock-down in macrophages caused an increased activation of NF-κB, which could be blocked by ammonium 1-pyrrolidinedithiocarbamate (PDTC). In addition, SOCS2 in macrophages also suppressed inflammation via limiting the activation of inflammasomes. Consistent with these, our BMT model also confirmed the SOCS2 function in macrophages during NASH.

**Interpretation:** Our data strongly indicate that SOCS2 plays a role in inhibiting inflammation and apoptosis via NF-κB and inflammasome signaling pathway in macrophages during NASH. Further studies are required to explore the potential preventive and therapeutic strategies of SOCS2 for this common liver disease.

## Introduction

Nonalcoholic fatty liver disease (NAFLD) is the hepatic consequence of metabolic syndrome, which is the most common cause of chronic liver disease in both industrialized and developing nations 1-3. A part of NAFLD ranges from simple steatosis to steatosis with progressive liver inflammation and fibrosis, a condition referred to as nonalcoholic steatohepatitis (NASH) 2, 4, 5. However, the mechanism of NASH pathogenesis remain unresolved.

Liver macrophages play a significant role in inflammation during NASH 6. It comprises subsets of different cell populations, including Kupffer cells and Infiltrating monocytes 7. Some reports have shown that in different stages of liver disease, resident Kupffer cells and freshly recruited monocyte-derived macrophages play a key part in the regulation of inflammation, fibrogenesis and fibrolysis 8. However, the function of liver macrophages is not studied well.

Since its discovery nearly 20 years ago, nuclear factor (NF)-κB has emerged as one of the most intensely studied eukaryotic transcription factors, chiefly because of its pleiotropic effects and its inducible pattern of expression that is subject to multi-level regulation 9, 10. Even though some studies show that the NF-κB signaling pathway regulates inflammation in NASH progression, the relationship is still unclear.

Thus, to study the mechanism of NASH, RNA-Seq was used to examine the mRNA level in macrophages in human NASH and Normal tissues. Notably, the suppressor of cytokine signaling 2 (SOCS2) was significantly decreased in NASH at transcription level. SOCS2 is a kind of adaptor protein that act as the substrate recognition subunits of Cullin/Ring ubiqiuitin ligases 11. Previous studies have found that hepatic SOCS2 deletion protected against hepatic steatosis but worsened insulin resistance in high-fat-diet-fed mice 12. In addition, SOCS2 has mainly been associated with growth hormone (GH) signaling and/or IGF-1 and is thereby involved in cell growth 13. Interestingly, SOCS2 can either act as an enhancer or suppressor of growth signaling depending on its expression level 13. Meanwhile, SOCS2 is involved in several inflammatory disorders and human NK cell function 14. Even though most of the role of SOCS2 has been revealed, its function in macrophages for inflammation during NASH progression is still unclear. Thus, understanding the function of SOCS2 in macorphages during NASH progression and the relationship between SOCS2, NF-κB and inflammation may attribute to understanding the mechanism of NASH pathogenesis and allow for an exploration into potential preventative and therapeutic strategies for NASH.

## Methods

### Patients

Human liver samples were obtained from random individuals who underwent liver biopsy or surgical resection at Jiangsu Province Hospital (Nanjing,China). The descriptions of characteristics of human samples have been provided in the **Table S1**. The tissues from liver biopsy were used in RNA-seq, making section, isolating protein and mRNA. The normal tissues were collected from the patients who received hepatic haemangioma surgery (n = 8) from 2018 to 2019, and the steatosis liver samples were collected from the eligible patients who received bariatric surgery (n = 36) from 2018 to 2020. Steatosis liver samples from individuals meeting any of the following criteria were excluded from the study: excessive alcohol consumption (>140 g for men or >70 g for women, per week), drug abuse, or viral infection (for example, infection with hepatitis B virus or hepatitis C virus). Assessment of NAFLD severity is based on the NAFLD Activity Score (NAS) that equals to a sum of unweighted scores of steatosis (0-3), lobular inflammation (0-3), and ballooning (0-2). Samples with Steatosis score of 0, Ballooning score of 0, Lobular inflammation score of 0 as No steatosis. Steatosis score of 1, Ballooning score of 1, Lobular inflammation score of 1 as simple steatosis. Steatosis score of 2-3, Ballooning score of 2-3, Lobular inflammation score of 2-3 as NASH. All procedures that involved human sample collection were approved by the tissue bank of Jiangsu Province Hospital, Nanjing Medical University (Nanjing, China). Informed consent for gene expression analysis of tissue was obtained from each patient before surgery, and the study was approved by our institutional ethics committee.

### NASH Mouse model

To establish NASH mice model, the mice mentioned in our experiment were euthanized after feeding with Western diet (Research Diets, Inc.) which contains 17 % Kcal protein, 17 % Kcal fat, 43 % Kcal Carbohydrate and 4.67 Kcal/g energy density, and supplemented with fructose in drinking water for 16 weeks after injected BM tranfecting with AVV-SCOS2^+^ at 8-week-old. The mice have been confirmed to develop the histopathological features of NASH, such as steatosis, inflammation, and fibrosis. Control mice were provided a standard chow diet and water *ad libitum*.

### Bone Marrow Transplantation (BMT) Mouse model

To overexpress SOCS2^+^ in macrophage *in vivo*, donor bone marrow (BM) cells from Wild-type were flushed from tibiae and femora in RPMI 1640 (BioWhittaker, Verviers, Belgium) and then transfecting with AAV-SOCS2^+^. After erythrocyte lysis using a buffered ammonium chloride solution, BM cells were resuspended in phosphatebuffered saline (PBS). After the isolation procedures, viability of BM cells was always more than 90% by trypan blue dye exclusion. C57BL/6 mice, used as recipients, received a total body irradiation (TBI) 16 hours before BMT. A 6- or 7-Gy single dose TBI was applied with a dose rate of 2.7 Gy/minute. Recipients then received a single intravenous (in a lateral tail vein) injection (300 mL) containing BM cells. No immunosuppressive agents were given.

### Oil Red O Staining

Oil-red-O staining was executed on the basis of a standard protocol. In brief, 0.5g Oil Red O powder (#CAS-1320-06-5, Sigma, Shanghai, China) was diluted in 100ml Isopropyl alcohol to make a Reserve liquid. Then, The working liquid was made by diluting and mixing Reserve liquid and ddH_2_O in proportion of 6 to 4. Next, frozen sections or cells were fixed with 4% paraformaldehyde and then incubated with 60% isopropanol for 1 minute. After incubating with the Working liquid for 10 minutes, the sections were washed with 75% alcohol for 5 seconds and then with ddH20 for 3 minutes. The nuclear were stained with hematoxylin. Photographs were taken with the microscope (OLYMPUS, BX43F) and software (XV Image Processing, 3.15.0.15404), and average values of integrated optical density (IOD) were obtained by analyzing five random felds per slide using Image-Pro Plus software v. 5.0. Every index was detected a minimum of three times.

### Macrophage isolation

Liver macrophages were isolated from WT mice fed with normal diet using the collagenase liver perfusion system. Briefly, the liver was perfused with a digestion buffer which contained collagenase for 5 minutes, chopped finely and filtered through a 70 µm mesh. After centrifugation at 50 g for 5 minutes, the hepatocyte fraction was discarded. Non-parenchymal cell fractions were layered on the top of Percoll gradients composed of 25% and 50% Percoll layers carefully. The liver macrophages fraction was isolated from between the two different Percoll gradient layers after centrifugation at 2300 rpm for 30 minutes. And the isolated liver macrophages were cultured with RPMI media containing 10% FBS for 1-2 days.

To isolate liver macrophages from human liver samples, 2-3 g fresh liver specimens were mechanically disrupted and dissociated with scissors (in 1-2 ml of cold complete RPMI). Dissociated tissue pieces were transferred into a 70μm strainer (placed into a 60-mm dish with 1-2 ml of cold complete RPMI) and further dissociated with the back of a 3-ml syringe. Cell suspension was filtered through 70 μm strainers. And the homogenate was spun down at 400 g for 5 min at 4 °C. Pellet was resuspended in 33% Percoll solution containing 10 U/ml heparin, and centrifuged for 15 min at 500 g at room temperature. Pellet was lysed with ammonium chloride potassium buffer. After incubation with Fc-blocker (2.4 G2; BD PharMingen, San Diego, CA), and stained with FITC-conjugated CD68 Ab with Cy5-streptavidin (ARG23069; Arigo biolaboratories Corp, Shanghai, China), the liver macrophages were sorted by Flow cytometry [FACS Aria II Cell Sorter (BD Biosciences)].

### RNA-seq

Human liver total RNA was extracted with PureLink/Trizol, according to the manufacturer's instruction. The amount, quality and composition of isolated RNA were analyzed by Nanodrop, Qubit 3.0 for total RNA and an Agilent 2100 Bioanalyzer for total RNA integrity. RNA purity was checked using the NanoDrop One/One C (Thermo Fisher Scientific, CA, USA). RNA concentration was measured by Qubit® RNA HS Assay Kit in Qubit®3.0 Fluorometer (Life Technologies, CA, USA). RNA integrity was assessed using the Agilent RNA 6000 Pico Kit of the Agilent Bioanalyzer 2100 system (Agilent Technologies, CA, USA). RNA-sequencing was performed by SHBio Inc., Shanghai, China. Sequencing libraries were generated using KAPA Stranded RNA-Seq Kit (KAPA, USA) following manufacturer's recommendations and index codes were added to attribute sequences to each sample. Libraries were pooled and sequenced on an Illumina X-ten PE150 platform. A minimum of 6G data were generated per sample.

### Flow cytometry

Flow cytometry was performed to check the apotosis of the cell arrocding to the manufacturer's instructions (A211; Vazyme Biotech Co, Nanjing, China). In brief, cells were collected and washed with PBS for 2 times, and Incubated with Annexin V-FITC and PI Staining Solution. Cells can be divided into three subgroups: living cells are double negative (Annexin V-FITC^-^/PI^-^); early apoptotic cells are Annexin V-FITC single positive (Annexin V-FITC^+^/PI^-^); Late apoptotic cells are double positive for Annexin V-FITC and PI (Annexin V-FITC^+^/PI^+^).

### Enzyme-Linked Immunosorbent Assay

ELISA was performed on cell supernatant collecting from RAW-Ctrl and RAW-SOCS2^+^/ RAW-shSOCS2 cells treated or not treated with FFAs using the Mouse IL-1β; TNFɑ; IL-6; IL-8; IL-17; IL-18 Platinum ELISA kit (shown in **Table S2**) per the manufacturer's instructions. All plants were detected by Spectrophotometer (Tecan Austria GmbH Untersbergstr.1A, REF:30086376) and software (Tecan, C25313).

### Cell Culture and Treatment

All the cells were purchased from Shanghai Institute for Biological Science (China). RAW264.7 cells were cultured in DMEM supplemented with 10% FBS, 100 U/ml penicillin, and 100 μg/ml streptomycin (Invitrogen, CA, USA) at 37 °C in a humidified incubator with 5% CO2.

For cell treatment, the FFAs mixture was prepared with oleic acid (OA; Sigma, O1257) and palmitic acid (PA; Sigma, P0500-10G) in a 2:1 ratio and used at a 0.5 mmol/L final concentration, for specified time periods (0, 6, 12, 18, 24 hours) to stimulate excessive uptake of FFAs.

### Ectopic Expression and Gene Silencing

For overexpression of SOCS2 were subcloned into the lentiviral vector PCDH-CMV-puro. The shRNA sequence specifically targeting SOCS2 was subcloned into the lentiviral vector pLKO.1-puro. Sequences of shRNA-SOCS2 are as followed:

- GCATCCGTTTCCACGACTTTCCTCGAGGAAAGTCGTGGAAACGGATGCTTTTTT, and;
- GCAAAGCTGGCACCAGAATTTCTCGAGAAATTCTGGTGCCAGCTTTGCTTTTTT.

Knockdown efficiencies are shown in **Figure 2B**. We selected shRNA specific for SOCS2 with the highest efficiencies for further experiments. Recombinant lentiviruses were produced by co-transfecting 293T cells with the lentiviral expression plasmid and packaging plasmids by Lipofectmine 3000 transfection reagent (Thermo Scientific) according to the manufacturer's protocol. Lentivirus was purified by ultracentrifugation and filtered for use.

### Cell transfection

The transfection of lentivirus stably expressing-SOCS2/shSOCS2 to RAW264.7 cells or primary marcophages were cultured in six-well dishes (2×10^5^/dish), and carried out without using polybrene for 24 hours.

### Western-bolt analysis

Proteins were extracted from tissues or cultured cells using RIPA containing phenylmethanesulfonylfluoride (Beyotime, Jiangsu, China). Then Equal amounts of proteins samples (100 µg) were separated using 7.5%/12.5% sodium dodecyl sulfate-polyacrylamide gel electrophoresis and transferred to polyvinylidene fluoride membrane. After blocking by 5% BSA in TBST, membranes were incubated with primary polyclonal antibodies. Secondary anti-rabbit and anti-mouse horseradish peroxidase-linked antibodies were purchased from WUHAN SANYING (Wuhan, Hubei, P.R.C). Blots were developed using electrochemiluminescence (ECL) reagent (Millipore, Billerica, MA, USA). Equal amounts of protein loading in each lane were confirmed using β-actin antibody. ImageJ software (NIH Image, Bethesda, MD, USA) was used to quantify the integrated density of the band. The related antibody was shown in **Table S3**.

### RNA isolation and quantitative real-time PCR (qRT-PCR)

Human tissue samples and all cells were homogenized in TRIzol (Invitrogen,Grand Island, NY, USA), and total RNA was isolated according to the manufacturer's suggested protocol: 1 μg of RNA was used for cDNA synthesis. To determine the relative level of cDNA, real-time PCR analyses were performed using an Applied Biosystems 7300 Detection System (Applied Biosystems®, CA). The real-time PCR reaction was performed according to the protocol of the SYBR® Premix Ex Taq™ kit (Takara, DRR041). Duplicate runs of each sample were normalized to β-actin to determine relative expression levels. All Primers were described in **Table S4**.

### Statistics

Data were analyzed using a Student's test or ANOVA followed by hoc t tests. All data are expressed as the mean ± SEM unless otherwise stated. *, **, *** and ****, indicate statistical significance with P <0.05, P < 0.01, P < 0.001 and P < 0.0001, respectively. Spearman's rank correlation coefficient analysis was applied to analyze the relationships between associated factors. Statistically non-significant results were labeled as n.s. where appropriate. All analyses were performed using GraphPad Prism 8 software.

## Results

### SOCS2 in macrophages was decreased during NASH progression and negative related to NASH level

**Figure 1:**
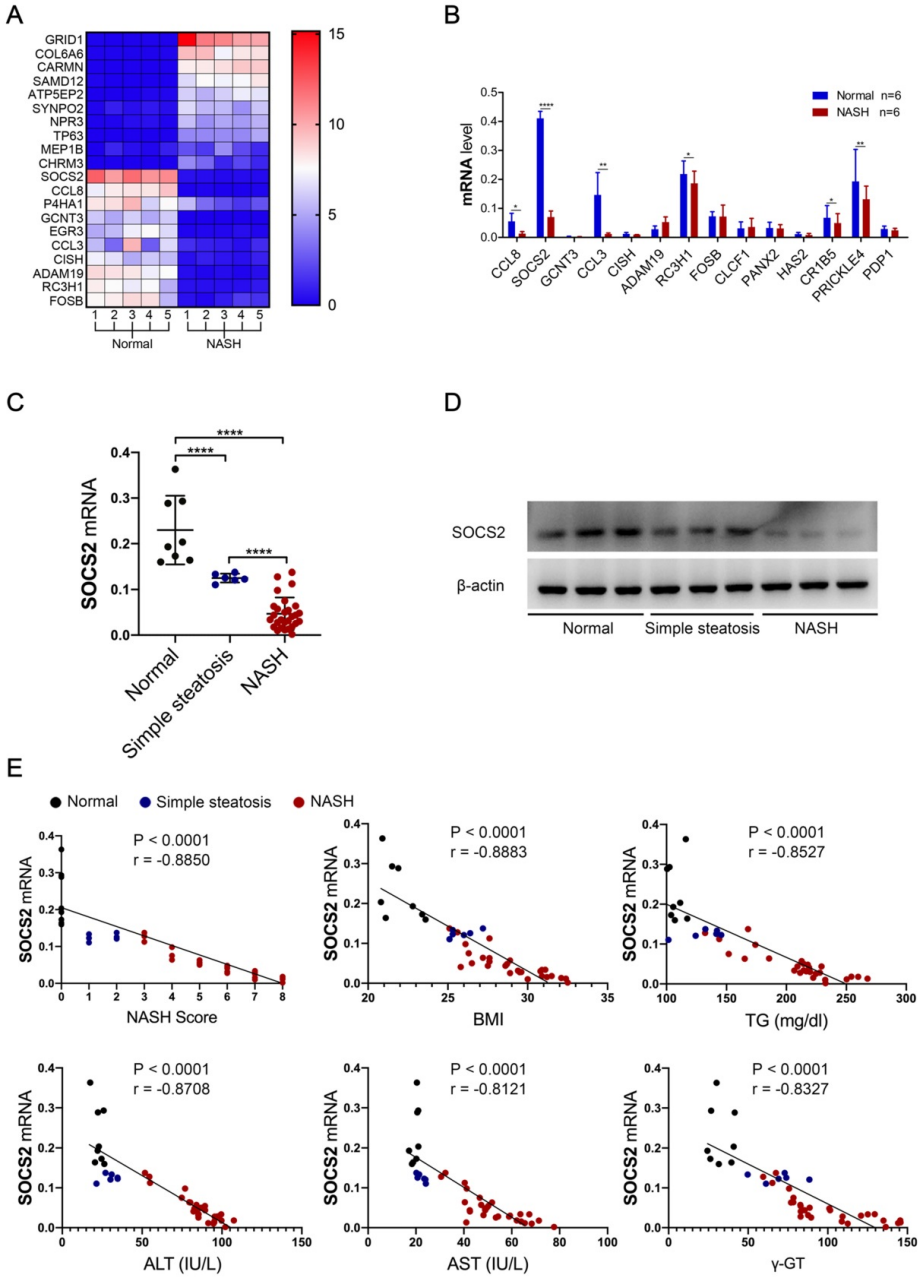
** SOCS2 in macrophages was decreased in macorphages during NASH progression and negative related to NASH level. A.** The heatmap from RNA-Seq of human liver macrophages with NASH (n=5) or Normal (n=5). **B.** The related markers expression were detect by RT-qPCR in human liver macrophages with NASH (n=6) or Normal (n=6). **C.** The related mRNA level of SOCS2 was detected by RT-qPCR in liver tissue macrophage samples of human subjects without steatosis, with simple steatosis and with NASH. **D.** Immunoblotting of SOCS2 and β-actin (loading control throughout) in liver tissue macrophage samples of human subjects without steatosis (n=3), with simple steatosis (n=3) and with NASH (n=3). **E.** Pearson comparison analyses of the correlation between SOCS2 mRNA level and NASH (r = -0.9471), BMI (r = -0.8542), serum TG content (r = -0.8908), serum γ-GT concentrations (r = -0.8406), serum AST concentrations (r = -0.9207) and serum ALT concentrations (r = -0.9112) (n = 153). P < 0.0001 for all of these correlations by Spearman′s rank correlation coefficient analysis.

To illuminate the mechanism of NASH, we detected the mRNA level in human NASH (n=5) and normal tissues (n=5) by RNA-Seq. The heat map analysis showed a novel gene, SOCS2, was significantly decreased in NASH group compared to Normal group (**Figure 1A, B**) (P<0.05). Next, to determine the clinical relevance of SOCS2 in NASH, we examined its expression in the liver tissue macrophage samples of human subjects without steatosis, with simple steatosis and with NASH. Consistent with our RNA-Seq result, SOCS2 mRNA and protein level were considerably lower in individuals with simple steatosis or NASH than in the nonsteatotic controls, and the NASH group had markedly lower SOCS2 expression than the simple steatosis group (**Figure 1C, D**) (P<0.001). Furthermore, SOCS2 mRNA levels in the liver were negatively correlated with the degree of NASH, as evidence of activity score, body mass index (BMI) and biochemical criterion (TG, γ-GT, ALT, AST) (**Figure 1E, Table S1**). These results demonstrate that SOCS2 expression is decreased in human steatohepatitis and inversely correlated to the NASH level (P<0.001).

### SOCS2 suppresses inflammation via limiting the activation of NF-κB signaling pathway

**Figure 2:**
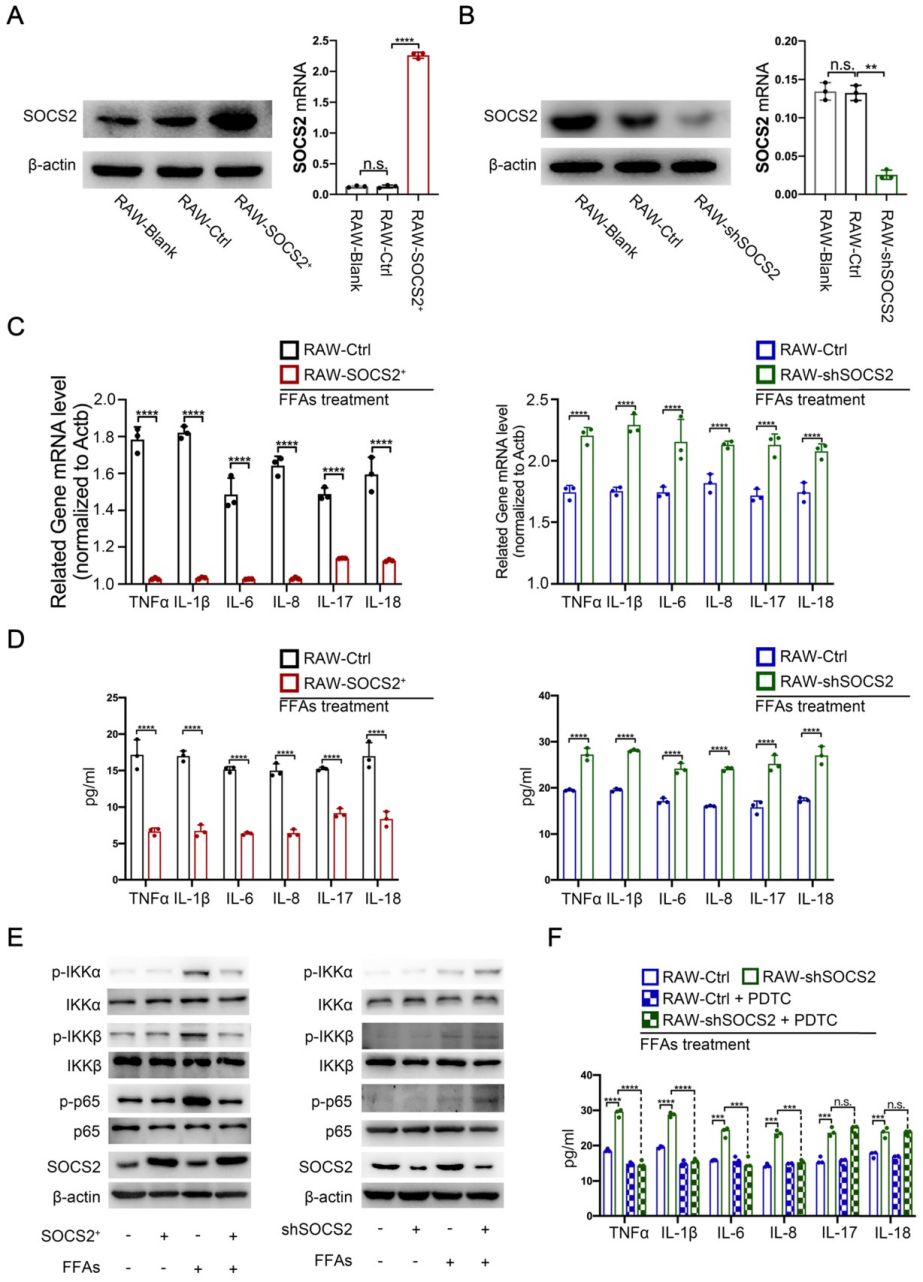
** SOCS2 suppresses inflammation via limiting the activation of NF-κB signaling pathway. A.** The related protein and mRNA level of SOCS2 was detected by Western blot and RT-PCR in RAW cell lines overexpreesing SOCS2 (RAW-Ctrl/ SOCS2^+^). **B.** The related protein and mRNA level of SOCS2 was detected by Western blot and RT-PCR in RAW cell lines knockdown SOCS2 (RAW-Ctrl/ shSOCS2). **C.** The related mRNA level of TNFα, IL-1β, IL-6, IL-8, IL-17, IL-18 was detected by RT-PCR in RAW-Ctrl or -SOCS2^+^ or -shSOCS2 treated with FFAs for 24 hr. **D.** The level of TNFα, IL-1β, IL-6, IL-8, IL-17, IL-18 was detected by ELISA in RAW-Ctrl or -SOCS2^+^ or -shSOCS2 treated with FFAs for 24 hr. **E.** Immunoblotting of phospho-IKKα, IKKα, phospho-IKKβ, IKKβ, phospho-P65, P65, SOCS2, and β-actin (loading control) in RAW-Ctrl or -SOCS2^+^ or -shSOCS2 treated with FFAs for 24 hr. **F.** The levels of IL-1β, TNFα, IL-6, IL-8, IL-17, and IL-18 in cell culture supernatants from RAW-Ctrl and RAW-shSOCS2 cells treated with or without FFAs and/or PDTC for 24 hr were detected by ELISA.

To confirm the role of SOCS2 in macrophages during NASH progression, we established the SOCS2-overexpressing and -konckdown cell lines in RAW 264.7 cell. The immunoblot and RT-qPCR analysis confirmed these (**Figure 2A, B**). Then, cells were stimulated with FFAs (free fatty acids in which the ratio of oleic acid: palmitic acid was at 2:1) to mimic the effect of *in vivo* metabolic stress. RT-qPCR and ELISA results demonstrated that SOCS2 overexpression caused decreased gene level of multiple inflammatory factors, including TNFα, IL-1β, IL-6, IL-8, IL-17 and IL-18 while SOCS2 knockdown caused a contrary results (**Figure 2C, D**). Cause NF-κB plays a significant role in inflammation 15, we detected the activation of NF-κB in these models *in vitro*. Results showed that overexpression of SOCS2 suppressed the activation of NF-κB signaling pathway, while SOCS2 knockdown also caused a contrary results (**Figure 2E**). Notably, the PDTC, an inhibitor of NF-κB, can block the SOCS2 knockdown-induced decreased level of inflammatory factors but not including IL-17 and IL-18 (**Figure 2F**). These data reveals that SOCS2 could suppresses inflammatory factors, including TNFα, IL-1β, IL-6, IL-8, via limiting the activation of NF-κB signaling pathway (**Figure 2F**). These results means that SOCS2 in macrophages suppresses inflammation by limiting the activation of NF-κB signaling pathway.

### SOCS2 in macrophages suppresses inflammation via limiting the activation of inflammasomes signaling pathway

**Figure 3:**
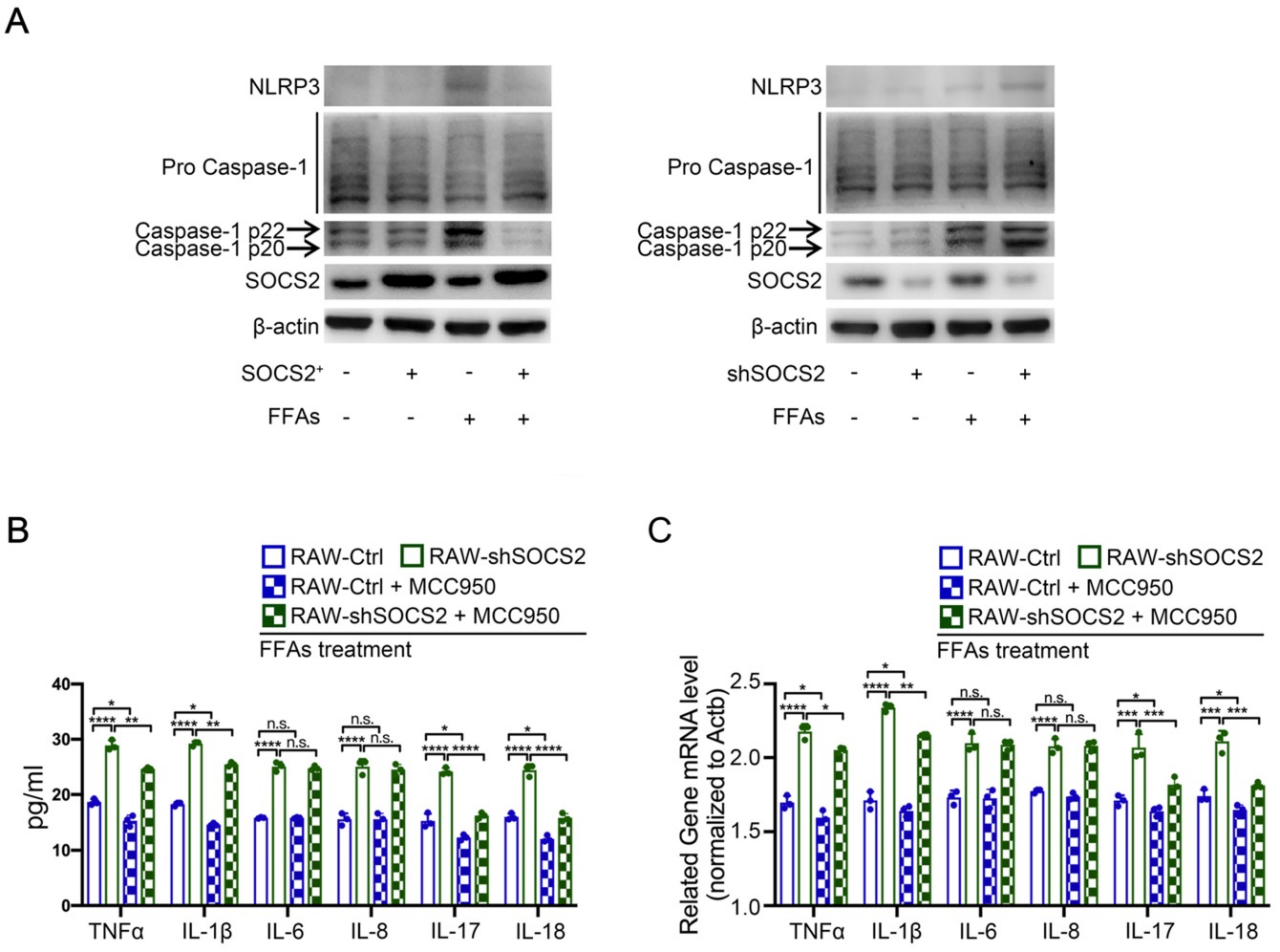
** SOCS2 in macrophages suppresses inflammation via limiting the activation of inflammasomes signaling pathway. A.** Immunoblotting of NLRP3, Pro-Caspase-1, Caspase-1 p20/p22 and β-actin (loading control) in RAW-Ctrl or -SOCS2^+^ or -shSOCS2 treated with FFAs for 24 hr. **B.** The levels of IL-1β, TNFα, IL-6, IL-8, IL-17, and IL-18 in cell culture supernatants from RAW-Ctrl and RAW-shSOCS2 cells treated with or without FFAs and/or MCC950 for 24 hr were detected by ELISA. **C.** The related mRNA level of TNFα, IL-1β, IL-6, IL-8, IL-17, IL-18 was detected by RT-PCR in RAW-Ctrl and RAW-shSOCS2 cells treated with or without FFAs and/or MCC950 for 24 hr were detected by ELISA.

Forgoing results showed that SOCS2 can suppresses inflammatory factors, including TNFα, IL-1β, IL-6, IL-8, via limiting the activation of NF-κB signaling pathway. However, there is a question that how SOCS2 regulates the level of IL-17 and IL-18. Considering inflammasomes are also involved in inflammation 16, the inflammasomes were detected in our FFAs-stress model *in vitro*. Immunoblot analysis suggested that SOCS2 overexpression causes a decreased level of NLRP3 and Caspase p20/22, while SOCS2 kncokdown causes an increased level of these markers (**Figure 3A**). In addition, RT-qPCR and ELISA results showed that SOCS2 knockdown-induced increased level of IL-17 and IL-18 can be notably blocked by MCC950-induced inhibiting the activation of NLRP3 (**Figure 3B, C**). These results means that SOCS2 in macrophages suppresses inflammation by two ways: i) limiting the activation of NF-κB signaling pathway; ii) limiting the activation of NLRP3.

### SOCS2 in macrophages suppresses apoptosis via limiting the activation of NF-κB signaling pathway

**Figure 4:**
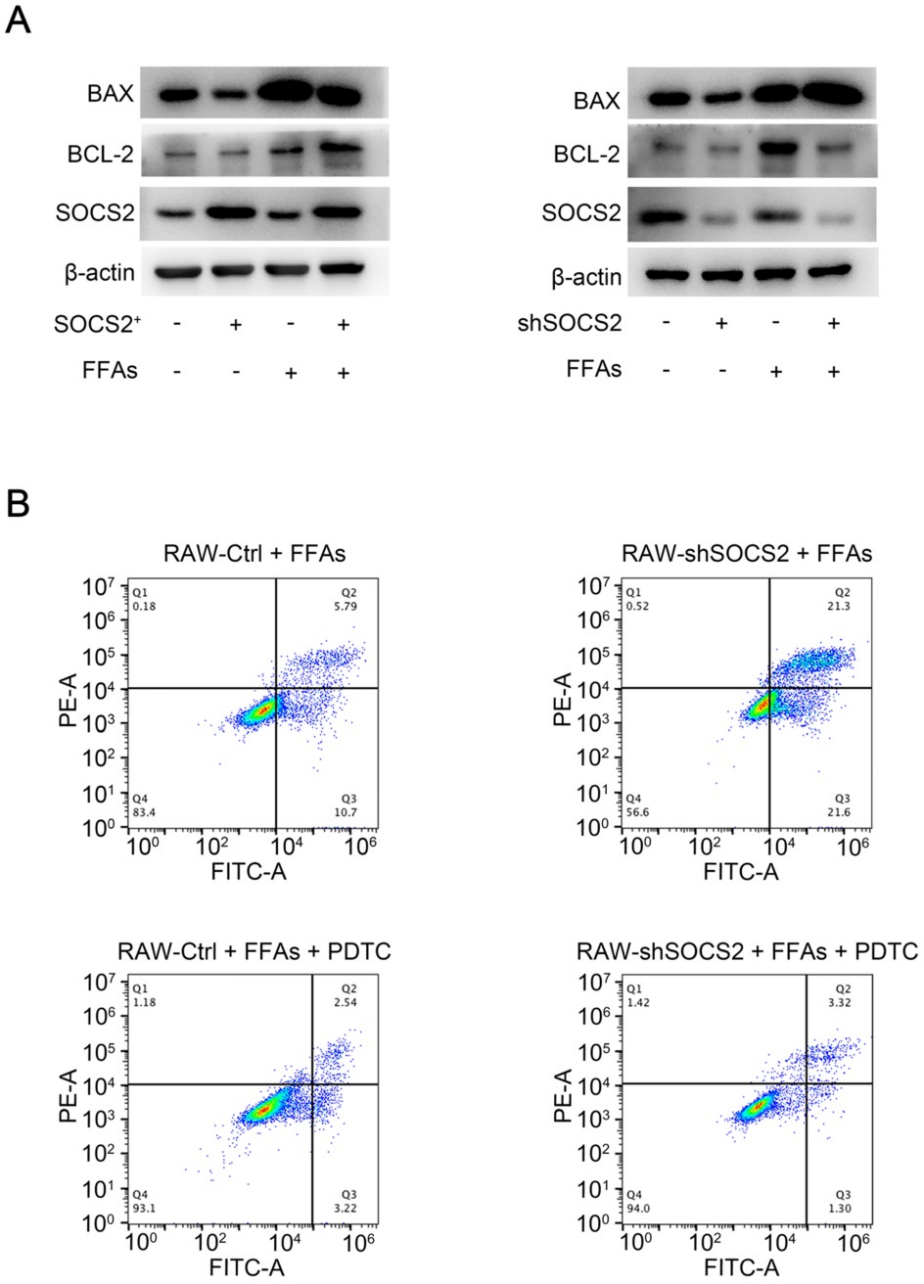
** SOCS2 in macrophages suppresses apoptosis via limiting the activation of NF-κB signaling pathway. A.** Immunoblotting of BAX, BCL-2, SOCS2 and β-actin (loading control) in RAW-Ctrl or -SOCS2^+^ or -shSOCS2 treated with FFAs for 24 hr. **B.** The level of apoptosis of RAW-Ctrl / SOCS2^+^ treated with FFAs or PDTC for 24 hr, detected by Flow cytometry.

Cell apoptosis is a pathological state during NASH 17, 18, we detected the target of cell apoptosis in our cell lines. As BCL-2 and BAX were major regulators of apoptosis, these two markers were detected in our FFAs-stress model *in vitro*. Results showed that SOCS2 overexpreesion leads to a decreased level of BAX and an increased level of Bcl-2, while SOCS2 knockdown causes a contrary result (**Figure 4A**). Considering NF-κB signaling pathway is upstream of BCL-2 and BAX, we revealed whether SOCS2 suppresses apoptosis via NF-κB signaling pathway, PDTC was used to blocking experiment. Cytometry results showed that SOCS2 knockdown caused an increased level of apoptosis, which can be completely blocked by PDTC *in vitro* (**Figure 4B**). Above all, these results demonstrate that hepatic SOCS2 can suppress apoptosis via limiting the activation of NF-κB signaling pathway.

### Overexpression of SOCS2 in macrophages restrains the development of NASH *in vivo*

**Figure 5:**
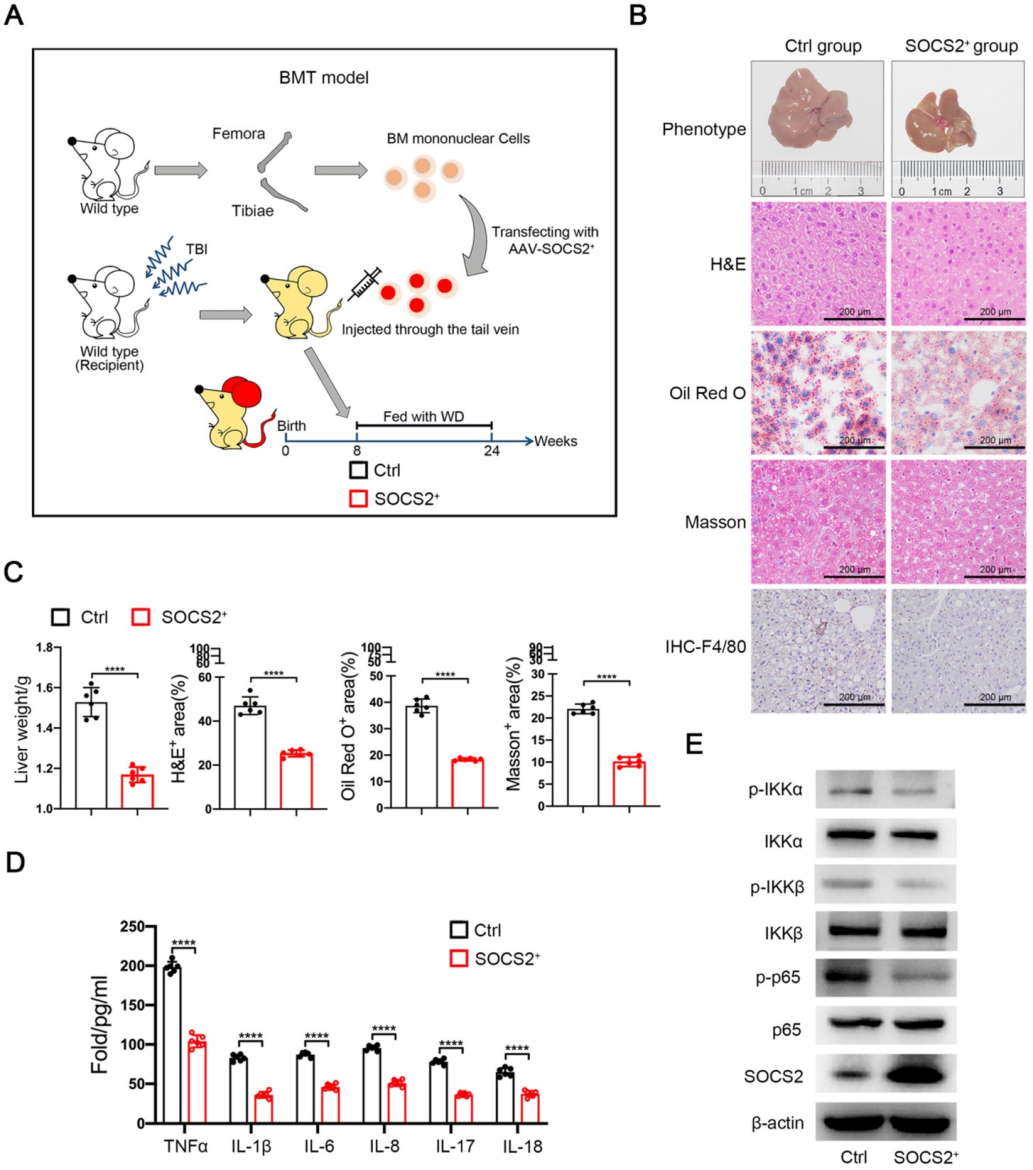
** Overexpression of SOCS2 in macrophages restrains the development of NASH *in vivo*. A.** The Western diet-induced NASH model in BMT mice (n = 6 per group). **B.** The liver from the mice described in **Figure 5A**. Liver sections were stained with Hematoxylin and Eosin (H&E), Oil-Red O, Masson and IHC (anti-F4/80), Original magnification, ×20. Bar = 200 μm. **C.** Quantitative analysis for **Figure 5B** of staining was shown. **D.** The level of serum TNFα, IL-1β, IL-6, IL-8, IL-17, IL-18 were determined in the mice described in **Figure 5B** by ELISA assay (n = 6 per group). **E.** Immunoblotting of phospho-IKKα, IKKα, phospho-IKKβ, IKKβ, phospho-P65, P65, SOCS2, and β-actin (loading control) in macrophage isolated from mice induced NASH (n = 6 per group).

To exclude molecular mechanism of SOCS2 in hepatocyte, we used the adeno-associated virus (AAV8) and BMT mice model to overexpress SOCS2 in wild-type mice macrophage and fed with Western diet (WD) supplemented with fructose in drinking water (**Figure 5A**). NASH model showed that SOCS2 overexpression alleviates the development of NASH compared with the controls, as evidence by a reduced whole body, increased hepatocyte vacuolation, steatosis, and fibrosis (**Figure 5B, C**). Meanwhile, SOCS2 overexpression also promoted activation of macrophages as evidence of an increased level of F4/80 (**Figure 5B**). In addition, the level of serum inflammatory factors was decreased in SOCS2^+^ group compared to Ctrl group (**Figure 5D**). To confirm the the relationship between SOCS2 and NF-κB signaling pathway, we isolated the macrophage from the mice induced NASH, results showed that overexpression of SOCS2 in mice suppressed the activation of NF-κB signaling pathway (**Figure 5F**), which consistent with the results *in vitro*. Above all, these data demonstrates that SOCS2 in macrophages suppresses NASH progression through limiting inflammation.

## Discussion

This study identifies that SOCS2 in macrophages plays a negative regulator in NASH progression. Nonalcoholic fatty liver disease (NAFLD) is the most popular chronic liver disease due to westernized diet and sedentary life style, which may evolve NASH due to inflammation and fibrosis 1-3. However, the mechanism of NASH pathogenesis remains unresolved. Our results demonstrate SOCS2 in macrophages suppresses inflammation and apoptosis via limiting the activation of NF-κB signaling pathway. In addition, SOCS2 in macrophages also suppresses inflammation via inhibiting inflammasome signaling pathway. Moreover, SOCS2 in macrophages was decreased in macorphages during NASH progression and negative related to NASH level.

SOCS2 is a kind of adaptor protein that act as the substrate recognition subunits of Cullin/Ring ubiqiuitin ligases 11. Previous studies have found that hepatic SOCS2 deletion protects against hepatic steatosis but worsens insulin resistance in high-fat-diet-fed mice 12. Our results are consistent with their study while we reveal that SOCS2 in macrophages not only suppresses inflammation but apoptosis. Meanwhile, SOCS2 is involved in several inflammatory disorders and human NK cell function 14. We also find that SOCS2 in macrophages suppresses inflammation not only via regulating NF-κB signaling pathway, but inflammasome signaling pathway.

As we known, one gene have a completely contrary function in different cells during disease 15, 19. For example, NF-kB can promote apoptosis and limit proliferation which suppresses HCC, while NF-kB in macrophages can enhance inflammation and promote the development of HCC. It is interesting that SOCS2 have a contrary function in hepatocytes and macrophages 20. The previous study shown SOCS2 aggravates hepatic steatosis via GH singaling pathway, however, our study found SOCS2 in marcophages limits apoptosis and inflammation. Considering inflammation and apoptosis plays a significant role during NASH 2, 3, 5, and SOCS2 can also inhibit insulin resistance 12, we think that SOCS2 in macrophages may play a major function, as an suppressor, for NASH progression and it in hepatocytes play a secondary role.

In our study, we choice Western diet (WD) supplemented with fructose in drinking water to induce NASH model in mice. Western diet (WD), which is high-fat, high-fructose and high-cholesterol, recapitulates features of the metabolic syndrome and NASH with progressive fibrosis 21. And this diet is mimic the NASH progression in human and is enough to induce NASH in mice, including lipid accumulation, inflammation and fibrosis. In addition, to reveal the role of SOCS2 in macrophages during NASH *in vivo*, we transplanted the primary macrophages overexpressing SOCS2 into Wild type mice treat with total body irradiation (TBI). This model, Bone Marrow Transplantation (BMT) 22 mice model, is a specific overexpress SOCS2 in all macrophages, which can clarify the role of SOCS2 in liver macrophages, exclude the function of SOCS2 in hepatocytes. Our *in vivo* results demonstrate that SOCS2 in macrophages can suppress NASH progression via limiting inflammation.

Inflammasomes and related inflammatory factors play an important role during NASH 23-25. During NASH progression, the activation of inflammasome leads to an increased level of inflammatory factors and push NASH progression 26. Our study find SOCS2 in macrophages suppresses inflammation not only by limiting NF-κB signaling pathway, but inflammasome signaling pathway.

Our study firstly identifies SOCS2 in macrophages plays a negative regulator for inflammation and apoptosis during NASH. SOCS2 suppresses inflammation via limiting NF-κB signaling pathway and inflammasome signaling pathway. Meanwhile, SOCS2 can also suppress apoptosis by inhibiting NF-κB signaling pathway. In addition, SOCS2 in macrophages is negatively related to NASH level. This model reveals SOCS2 in macrophages is a core of the network among Inflammasomes, inflammation and NASH, and provides a novel molecular pathogenesis of NASH which could be applicable to human NASH potential prevention and therapeutic strategies in future.

## Research in Context

### Evidence before this study

Nonalcoholic fatty liver disease (NAFLD) is the hepatic consequence of metabolic syndrome, which is the most common cause of chronic liver disease in both industrialized and developing nations. Suppressor of cytokine signaling 2 (SOCS2) is one of classic negative regulators of cytokine signaling, which has recently been described as anti-inflammatory mediators. However, the role of SOCS2 in macrophages during NASH progression and the relationship among SOCS2, inflammation, apoptosis and NASH is largely unknown.

### Added value of this study

We examined the relationship among SOCS2 in macrophages, inflammation, apoptosis and NASH in clinical samples, experimental model of NASH *in vivo* and vitro. We showed that SOCS2 overexpression in macrophages suppresses NASH development via limiting inflammation and apoptosis, while SOCS2 knock-down in macrophages aggravates NASH progression by promoting inflammation and apoptosis. The mechanism is related to NF-κB and inflammasomes signaling pathway.

### Implications of all the available evidence

SOCS2 plays an inhibitor of inflammation and apoptosis via NF-κB signal pathway and inflammasome signal pathway in macrophages during NASH. This model reveals SOCS2 in macrophages is a core of the network among Inflammasomes, inflammation and NASH, and provides a novel molecular pathogenesis of NASH which could be applicable to human NASH potential prevention and therapeutic strategies in future.

## Supplementary Material

Supplementary tables.
