# Supplementary material for: SOCS2 Suppresses Inflammation and Apoptosis during NASH Progression through Limiting NF-κB Activation in Macrophages
Source: Int J Biol Sci. 2021 Oct 11;17(15):4165–75. doi: 10.7150/ijbs.63889 (PMC8579457; doi:10.7150/ijbs.63889)
Supplement: Supplementary file 1 — Supplementary tables. [file ijbsv17p4165s1.pdf]

Table S1: The related chracteristics of liver tissue samples of human subjects without steatosis, with simple steatosis and with NASH

Clinical Features	No steatosis n=8	Simple steatosis n=6	NASH n=28
Age (years)	39.55±11.14	35.50 ±10.47	34.03 ± 9.08
Gender			
Male	4	4	15
Female	4	2	13
Body mass index (kg/m2)	22.00±1.13	25.88±0.81	28.93±2.21
Insulin (U/L)	5.2 ± 2.5*	23.20 ± 12.58	33.26 ± 19.65
Triglycerides (mg/dL)	108.02±6.26	131.25±16.48	209.89±34.03
ALT (U/L)	22.82±2.92	29.86±5.02	85.89±13.87
AST (U/L)	19.70±1.42	22.31±1.75	52.43±11.92
γ-GT (U/L)	32.60±7.03	69.25±13.06	100.02±26.30
Steatosis (%)			
Grade 0	8 (100%)		
Grade 1		6 (100%)	
Grade 2			7(25%)
Grade 3			21 (75%)
Ballooning (%)			
Grade 0	8 (100%)		
Grade 1		6 (100%)	
Grade 2			7 (25%)
Grade 3			21(75%)
Lobular inflammation (%)			
Grade 0	8 (100%)		
Grade 1		6 (100%)	
Grade 2			7 (25%)
Grade 3			21 (75%)

Table S2

Kit	
Mouse IL-1 beta Platinum ELISA kit	R&D Systems, MLB00C
Mouse TNFα Platinum ELISA kit	R&D Systems, MTA00B
Mouse IL-6 Platinum ELISA kit	R&D Systems, M6000B
Mouse IL-8 Platinum ELISA kit	R&D Systems, D8000C
Mouse IL-17 Platinum ELISA kit	R&D Systems, M1700
Mouse IL-18 Platinum ELISA kit	R&D Systems, 7625

Table S3

Antibody		
IKKα	CST	2628S
Phospho-IKKα	CST	2697S
IKKβ	abcam	ab55404
P65	CST	8242S
Phospho-P65	CST	3033S
SOCS2	CST	2779S
NLRP3	abcam	ab214185
Caspase-1-P20	Proteintech	22915-1-AP
β-actin	Proteintech	66009
Bax	CST	14796S
Bcl-2	CST	3498S

Table S4

Gene	Species	Forward Primer	Reverse Primer
**IL-1b**	Mus	GCAACTGTTCCTGAACTCAACT	ATCTTTTGGGGTCCGTCAACT
**TNFa**	Mus	CCCTCACACTCAGATCATCTTCT	GCTACGACGTGGGCTACAG
**IL-6**	Mus	CCAAGAGGTGAGTGCTTCCC	CTGTTGTTCAGACTCTCTCCCT
**IL-8**	Mus	CAAGGCTGGTCCATGCTCC	TGCTATCACTTCCTTTCTGTTGC
**IL-17**	Mus	TTTAACTCCCTTGGCGCAAAA	CTTTCCCTCCGCATTGACAC
**IL18**	Mus	CAAGGCTGGTCCATGCTCC	TGCTATCACTTCCTTTCTGTTGC
**SOCS2**	Mus	AGTTCGCATTCAGACTACCTACT	TGGTACTCAATCCGCAGGTTAG
**β-actin**	Mus	GGCTGTATTCCCCTCCATCG	CCAGTTGGTAACAATGCCATGT
**SOCS2**	Ho	TTAAAAGAGGCACCAGAAGGAAC	AGTCGATCAGATGAACCACACT
**IL-1b**	Ho	ATGATGGCTTATTACAGTGGCAA	GTCGGAGATTCGTAGCTGGA
**TNFa**	Ho	CCTCTCTCTAATCAGCCCTCTG	GAGGACCTGGGAGTAGATGAG
**IL-6**	Ho	ACTCACCTCTTCAGAACGAATTG	CCATCTTTGGAAGGTTCAGGTTG
**IL-8**	Ho	ACTGAGAGTGATTGAGAGTGGAC	AACCCTCTGCACCCAGTTTTC
**IL-17**	Ho	GAGCCCCAAAAGCAAGAGGAA	TGCGGGCATACGGTTTCATC
**IL18**	Ho	TCTTCATTGACCAAGGAAATCGG	TCCGGGGTGCATTATCTCTAC
**CCL8**	Ho	TGGAGAGCTACACAAGAATCACC	TGGTCCAGATGCTTCATGGAA
**GCNT3**	Ho	TCTGGGCTGCTATATGCTGC	GTTGATAGACCTCTTTGCTGGAA
**CCL3**	Ho	AGTTCTCTGCATCACTTGCTG	CGGCTTCGCTTGGTTAGGAA
**CISH**	Ho	GAACTGCCCAAGCCAGTCAT	GCTATGCACAGCAGATCCTCC
**ADAM19**	Ho	GGGAGCCTGGATGGACAAG	AGCTTTGAGTGGATGCTTTTCTC
**RC3H1**	Ho	TCCACAATGGACGGATTTCCT	AACCCAAACTGATGGGCTTTC
**FOSB**	Ho	GCTGCAAGATCCCCTACGAAG	ACGAAGAAGTGTACGAAGGGTT
**CLCF1**	Ho	TTTCAACGAGCCAGACTTCAAC	GAGGCCACGCAAGTAACACA
**PANX2**	Ho	TTCGCCGCCATCATGTACG	GGTAACAGTTGTCGATCTCCTG
**HAS2**	Ho	TCCTGGATCTCATTCCTCAGC	TGCACTGAACACACCCAAAATA
**PRICKLE4**	Ho	CTGCTTCGAGAACCGCTACTC	GTCCCTTCCTTCAGTTCGGTC
**PDP1**	Ho	CCTGCAAATGCACCCATTGAG	CTGCCTGGGAACAAGCACAA
**β-actin**	Ho	CATGTACGTTGCTATCCAGGC	CTCCTTAATGTCACGCACGAT
